# Evaluation of Monocular Treatment for Meibomian Gland Dysfunction with an Automated Thermodynamic System in Elderly Chinese Patients: A Contralateral Eye Study

**DOI:** 10.1155/2016/9640643

**Published:** 2016-12-27

**Authors:** Yinying Zhao, Jialu Xie, Junhua Li, Yana Fu, Xiaolei Lin, Shangrong Wang, Jiling Ma, Yune Zhao

**Affiliations:** ^1^School of Ophthalmology and Optometry, Eye Hospital, Wenzhou Medical University, Wenzhou, Zhejiang, China; ^2^Key Laboratory of Vision Science, Ministry of Health, Wenzhou, Zhejiang, China

## Abstract

*Purpose.* To investigate the safety and efficacy of monocular treatment for elderly Chinese patients with meibomian gland dysfunction (MGD) with an automated thermodynamic system.* Methods.* This study was a prospective, examiner-masked, contralateral eye clinical trial. The eye perceived by the patient to be worse (test eye) received a 12-minute LipiFlow treatment, while the other eye served as control. All patients were examined before treatment and one week, one month, and three months after treatment. Clinical parameters included dry eye symptoms, lipid layer thickness (LLT), partial blink (PB) ratio, invasive tear breakup time (ITBUT) and cornea staining, Schirmer I test, meibomian glands yielding liquid secretion (MGYLS), and meibomian gland dropout.* Results.* A total of 29 patients were examined during the three-month follow-up. At each posttreatment visit, they had a significant reduction in dry eye symptoms accompanied by an increase of ITBUT and MGYLS and a reduction in corneal staining compared with the baseline parameters. There was a significant improvement in MGYLS and ITBUT in the test eye compared with the control eye. Other clinical parameters were not statistically significant.* Conclusion.* LipiFlow is an effective treatment for patients with MGD. Monocular treatment with LipiFlow may be a cost-effective treatment option to those afflicted with MGD in the developing world.

## 1. Introduction

In 2011, the International Workshop on Meibomian Gland Dysfunction announced a new definition of MGD: “a chronic, diffuse abnormality of the meibomian glands, commonly characterized by terminal duct obstruction and/or qualitative/quantitative changes in the glandular secretion. It may result in alteration of the tear film, symptoms of eye irritation, clinically apparent inflammation, and ocular surface disease [1].”

Today, numerous clinical approaches are being undertaken to relieve meibomian gland obstruction. The most effective and well-known treatment is warm compress combined with lid massage, which heats the glands and alleviates gland obstruction [2, 3]. However, for this treatment to be effective, an intensive treatment regimen is required to be performed by the patient, making patient compliance low [4, 5].

A promising new instrument called LipiFlow (TearScience Inc., Morrisville, NC) has been specifically designed to partially or possibly completely alleviate meibomian gland obstruction. This instrument combines the benefits of both heat therapy and physical expression while accurately controlling the temperature, pressure, and technique.

Several studies [6–8] have reported the improvement in meibomian gland secretion after one 12-minute treatment by LipiFlow and the effects last up to one year. Tear film breakup time (TBUT) and symptom scores also showed improvement compared with traditional therapies [9–11]. Previous studies were performed with concurrent binocular therapy, which may be cost prohibitive in developing countries and may introduce statistical errors in the analysis of the study. This is the first study to evaluate the effect of monocular therapy by LipiFlow. Overall, the objective of this study was to determine the efficacy of monocular treatment with LipiFlow, with the contralateral eye as a control, in elderly Chinese patients with MGD.

## 2. Patients and Methods

This prospective, examiner-masked, clinical trial was performed at Wenzhou University, Zhejiang, China, and was approved by the Institutional Review Board/Ethics Committee of Wenzhou Medical University. Informed consent to participate in this research study was obtained from each patient. Practices and researches were conducted in accordance with the tenets of the Declaration of Helsinki. The study was registered at www.clinicaltrials.gov and the clinical trial accession number is NCT02481167.

### 2.1. Patients

Patients who completed a comprehensive baseline ocular surface evaluation were enrolled in accordance with the inclusion criterion. Eligible patients were recruited. The eye which the patient perceived as worse was selected as the test eye. The test eye received a 12-minute LipiFlow treatment. The contralateral eye served as the control eye. Data from both eyes were recorded before treatment as baseline and were reexamined at one week, one month, and three months posttreatment. Patients who met the following criteria were eligible for the study.

#### 2.1.1. Inclusion Criteria

1. Age from 55 to 75 years old
2. Reported dry eye symptoms with a standard patient evaluation for eye dryness (SPEED) score greater than 6 at the baseline
3. Meibomian gland atrophy less than fifty percent
4. Evidence of functional meibomian gland (clear liquid secretion) number being 6 or less
5. Informed consent given to participate in the study
6. Ability to complete an initial evaluation and the follow-up visits after treatment (one week, one month, and three months)

#### 2.1.2. Exclusion Criteria

1. Ocular trauma, surgery, or active infection in either eye within three months of the baseline examination
2. Being determined to have dry eye symptoms that were not secondary to MGD, such as a systemic autoimmune disease
3. Abnormalities that may potentially affect the integrity of the cornea or lid function in either eye
4. Involvement in other ophthalmic drug or medical device clinical trials within 30 days of the baseline examination
5. Treatment with lacrimal plugs or canaliculoplasty in either eye within three months of the baseline examination or the potential of requiring new treatment during the follow-up period

### 2.2. Methods

Before the initial clinical examination, patients were asked to report their clinical history and general physical condition. Meibography was only recorded at the baseline and three-month visit. The following clinical tests were performed at each follow-up visit in this order: dry eye questionnaire, best corrected visual acuity (BCVA), Lipiview Interferometer examination, slit-lamp examination, TBUT, corneal staining, Schirmer I test, and extrusion of meibomian glands. In order to control for examiner variability, the same experienced masked clinical investigator conducted all examinations. During the follow-up period, ocular lubricants and warm compress treatments were not restricted for humanitarian reasons.

### 2.3. Investigated Parameters 

#### 2.3.1. Dry Eye Symptoms

Two questionnaires, Standard Patient Evaluation of Eye Dryness (SPEED) [12] and the Ocular Surface Disease Index (OSDI), were used to assess dry eye symptoms.

#### 2.3.2. Lipid Layer Thickness (LLT) and Partial Blink (PB)

Lipiview Interferometer (TearScience Inc., Morrisville, NC) was used to determine the lipid layer thickness (LLT) and blink pattern (BP). A 20-second video of each eye was captured to record the interference pattern of the tear film. The software uses an algorithm which converts the value in interferometric color units (ICUs) into nanometers of lipid layer thickness (LLT) (1 ICU approximately reflects 1 nm of the LLT). Additionally, partial blinks (PB) and total blinks were also recorded. The partial blink (PB) ratio equals the number of partial blinks/the number of total blinks.

#### 2.3.3. Invasive Tear Breakup Time (ITBUT) and Corneal Staining [13]

To evaluate the TBUT and corneal staining, strips of fluorescein sodium (containing 1.0 mg fluorescein sodium) (Jing Ming New Technological Development Co., Ltd., Tianjin, China) were used. To perform this, patients were told to look up while the tip of the strip quickly touched the inferior conjunctiva. ITBUT was calculated by calculating the average of three consecutive breakup times, as determined manually by a stopwatch.

Three minutes after fluorescein instillation slit-lamp biomicroscopy cobalt blue illumination was used to evaluate corneal staining. The cornea was divided into four quadrants (supertemporal, inferotemporal, supernasal, and inferonasal). Superficial punctate keratopathy in the cornea was scored from 0 to 3 in each quadrant: 0, no staining in the cornea; 1, <5 punctuate staining; 2, >5 punctuate staining but <10; and 3, >10 punctuate staining. A sum of the staining scores ranged from 0 to 12.

#### 2.3.4. Schirmer I Test without Anesthesia

The Schirmer I test was performed without anesthesia. Schirmer Tear Test Strips (Jing Ming New Technological Development Co., Ltd., Tianjin, China) were placed between the lateral and middle third of the lower eyelid and patients were instructed to close their eyelids for five minutes.

#### 2.3.5. Meibomian Gland Assessment

To assess meibomian gland function, the number of functional glands was counted. A gland was considered functional if it yielded a clear liquid secretion, which represents both the quantity and the quality of meibomian gland secretions. The number of meibomian gland orifices was quantified using the Meibomian Gland Evaluator (TearScience Inc.). Using this handheld instrument, a defined pressure (a consistent force of 1.25 g/mm^2^, similar to that experienced with a deliberate or forced blink) was applied to the nasal, central, and temporal regions of the lower eyelid. Each region contained five consecutive meibomian gland orifices. A total of 15 glands were evaluated along the lower eyelid margin. Finally, the number of glands secreting clear liquid was counted.

#### 2.3.6. Meibomian Gland Dropout [14]

The Keratograph 5M (Oculus, Wetzlar, Germany) is an advanced corneal topographer that can be used for meibography. Infrared meibography with high-contrast images of the meibomian glands was employed to observe upper and lower everted eyelids of each eye. A meiboscore was given according to calculations assigned by ImageJ software (Ver. 1.50b, National Institutes of Health, USA) for each eyelid corresponding to the percentage of meibomian gland affected by atrophy. The overall percentage of abnormalities of each lid was described as 0% (grade 0), less than 33% (grade 1), 33 to 67% (grade 2), or greater than 67% (grade 3) [15]. The higher the score, the more severe the meibomian atrophy.

#### 2.3.7. Additional Clinical Parameters

Several additional parameters were assessed to evaluate the safety of LipiFlow instrument. These included adverse events (during the study period) and an ocular health exam (eyelid marginal hyperemia and pachyblepharosis).

### 2.4. Statistical Analysis

Statistical analysis was performed using SPSS 19.0 for Microsoft Windows (Chicago, Illinois, USA). To compare the baseline and posttreatment outcomes for the test group, paired two-tailed *t*-test was used where the normal distribution was confirmed using the Kolmogorov–Smirnov test, *p* > 0.05). The Wilcoxon signed-rank test was used for nonparametric distributed data (Kolmogorov–Smirnov test, *p* < 0.05). A repeated measures analysis of variance (ANOVA) test was performed for comparison of test group and control group at all time points. A difference was considered statistically significant if a value of *p* = 0.05 was reached.

## 3. Results

Thirty subjects were initially enrolled; however, 29 subjects (58 eyes, 56.90 ± 7.07 years old, 20 (69%) women and 9 (31%) men) completed the study. One patient enrolled in the study had to be excluded after the baseline examination due to treatment for meningeal gliomatosis.

### 3.1. Comparison of the Test Eye at Baseline and after Treatment

#### 3.1.1. Subjective Symptoms

**Figure 1:**
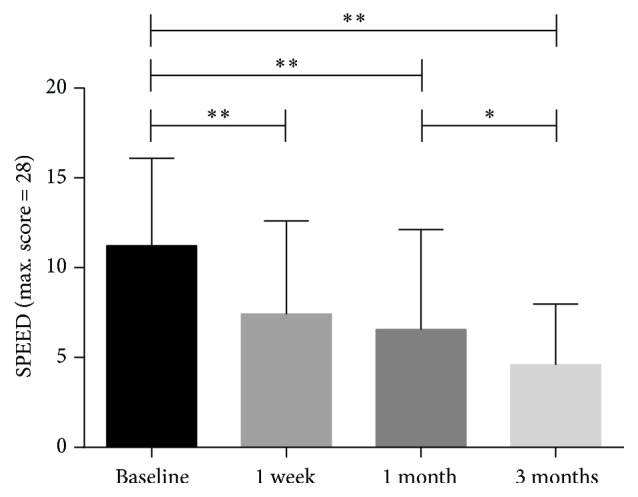
The mean value of test-eye Standard Patient Evaluation of Eye Dryness (SPEED) score measured at baseline, at one week, at one month, and at three months (^*∗*^*p* < 0.05, ^*∗∗*^*p* < 0.001).

**Figure 2:**
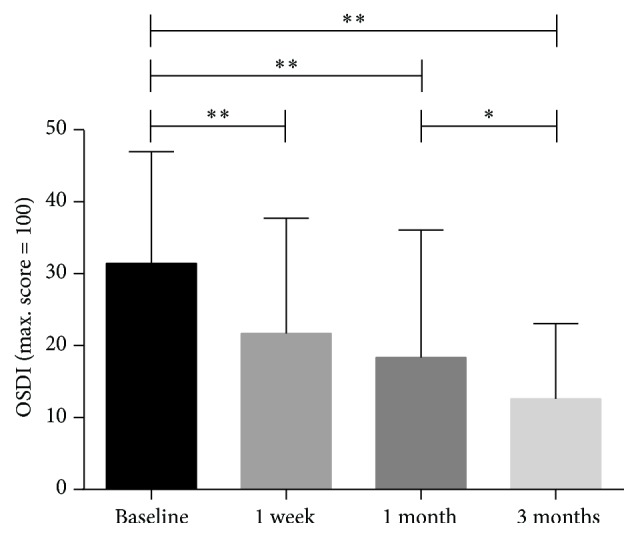
The mean value of test-eye Ocular Surface Disease Index (OSDI) at baseline, at one week, at one month, and at three months (^*∗*^*p* < 0.05, ^*∗∗*^*p* < 0.001).

There was a significant improvement in SPEED scores at the one-week visit (7.43 ± 5.18, *p* < 0.0001) compared with the baseline scores (11.22 ± 4.87) and this improvement was maintained at the one-month time point (6.55 ± 5.57, *p* < 0.001). Similarly, at the three-month time point (4.59 ± 3.40, *p* < 0.001), the observed improvement was still significant. However, the difference between the one-week and the one-month visits was not statistically significant (*p* = 0.343, *p* > 0.05). SPEED scores continued to decrease from the one-month visit to the three-month visit (*p* = 0.017, *p* < 0.05) (Figure 1). A significant improvement of dry eye symptoms was observed in both one-week and one-month follow-ups. Also, a further improvement was found in the three-month follow-up. Results of OSDI score were consistent with SPEED scores (Figure 2).

#### 3.1.2. Changes of Tear Film

**Figure 3:**
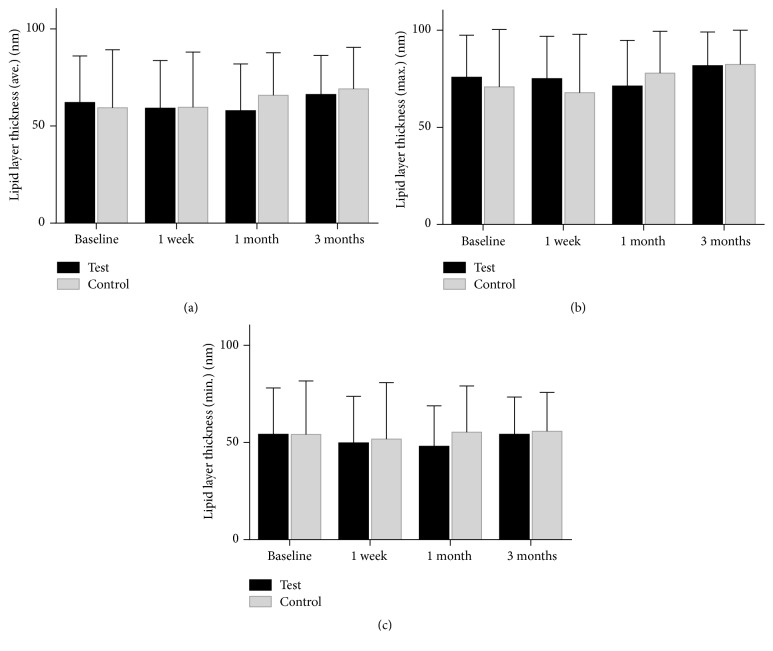
The mean value of test-eye lipid layer thickness (LLT) at baseline, at one week, at one month, and at three months. (a) The average of LLT. (b) The maximum of LLT. (c) The minimum of LLT.

**Figure 4:**
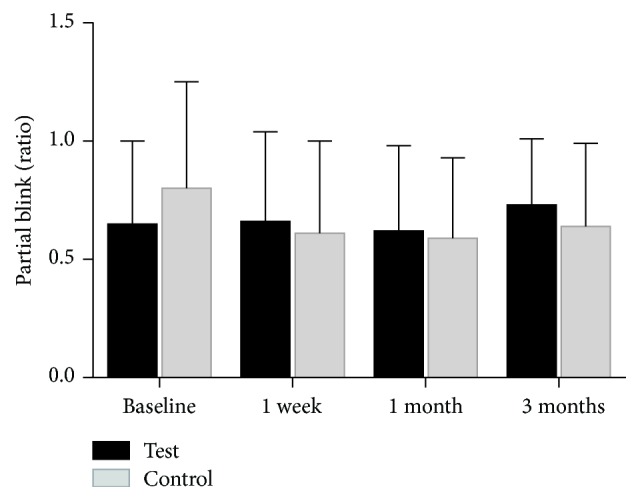
The mean value of test-eye partial blink (PB) ratios at baseline, at one week, at one month, and at three months.

No statistically significant change in the average, maximum, or minimum in the lipid layer thickness (LLT) was observed from preprocedure time point to one week, one month, and three-month postprocedure visits (Figure 3). Partial blink (PB) ratios held a similar trend as no statistical difference between the initial evaluation and any of the follow-up visits after treatment was recorded (Figure 4).

**Figure 5:**
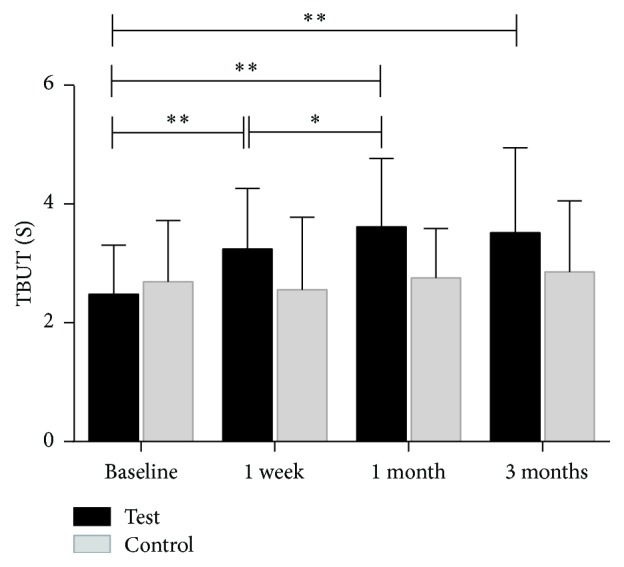
The mean value of test-eye tear breakup time (TBUT) measured at baseline, at one week, at one month, and at three months (^*∗*^*p* < 0.05, ^*∗∗*^*p* < 0.001).

A statistically significant increase in TBUT from the preprocedure time point (2.48 ± 0.83) to the one-week postprocedure visit (3.24 ± 1.02; *p* = 0.003) was reported. This increase remained significant at the one-month (3.62 ± 1.15; *p* = 0.001) and the three-month (3.52 ± 1.43; *p* = 0.004) postprocedure visits. The difference between one-week and one-month visits was also statistically significant (*p* = 0.033, *p* < 0.05). However, the difference between one-month and three-month visits was not statistically significant (*p* = 0.687, *p* > 0.05) (Figure 5). An increase of TBUT was observed at all follow-up visits; however, further improvement was not observed at the three-month follow-up visit.

**Figure 6:**
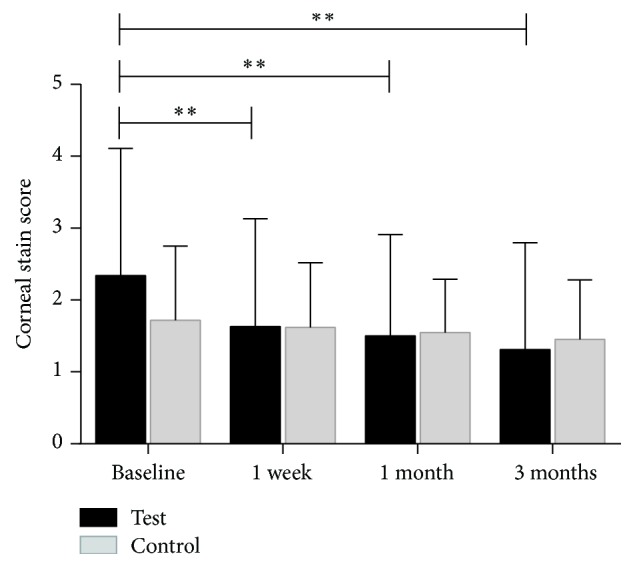
The mean value of test-eye cornea staining score measured at baseline, at one week, at one month, and at three months (^*∗*^*p* < 0.05, ^*∗∗*^*p* < 0.001).

Total score of the four corneal regions examined was on a scale of 0 to 12 at the baseline, one-week, one-month, and three-month examinations. The total sodium fluorescein staining scores of the corneal epithelium were reduced from baseline (2.34 ± 1.77) to one week (1.63 ± 1.50, *p* = 0.002). There was a statistically significant reduction in the fluorescein staining scores when comparing baseline visit data to one-month (1.50 ± 1.41, *p* < 0.0001) and three-month (1.31 ± 1.49, *p* < 0.0001) posttreatment visit data (Figure 6).

**Figure 7:**
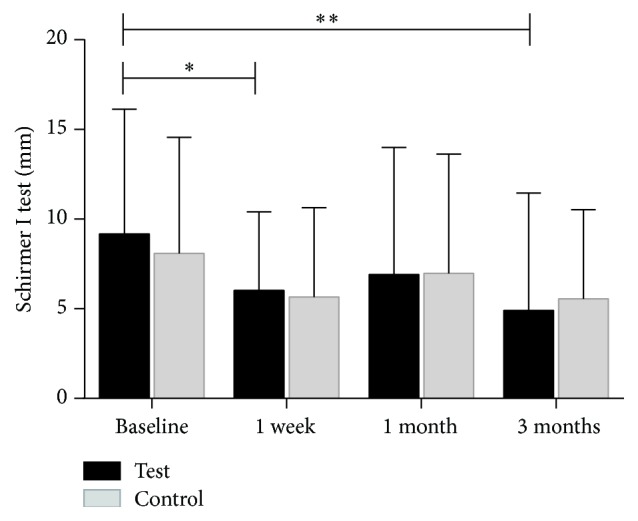
The mean value of test-eye Schirmer I test without anesthesia measured at baseline, at one week, at one month, and at three months (^*∗*^*p* < 0.05, ^*∗∗*^*p* < 0.001).

There was a statistically significant decrease in Schirmer I test without anesthesia from the preprocedure time point (8.17 ± 6.96) compared to the one-week postprocedure visit (6.03 ± 4.38; *p* = 0.040, <0.05). A nonsignificant decrease was observed at the one-month postprocedure visit (6.90 ± 7.09; *p* = 0.086), However, at the three-month postprocedure visit, a significant decrease compared to baseline (4.90 ± 6.56; *p* = 0.004, <0.05) was observed. The difference between one-week and one-month visits was not statistically significant (*p* = 0.680, >0.05). The difference between one-month and three-month visits was also not statistically significant (*p* = 0.120, *p* > 0.05) (Figure 7).

### 3.2. Improvements of Meibomian Gland Function

#### 3.2.1. Meibomian Glands Yielding Liquid Secretion (MGYLS)

**Figure 8:**
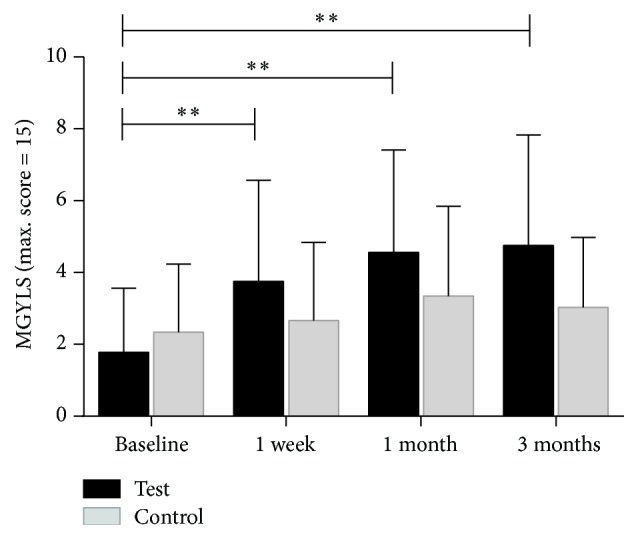
The mean value of test-eye meibomian glands yielding liquid secretion (MGYLS) measured at baseline, at one week, at one month, and at three months (^*∗∗*^*p* < 0.001).

A significant increase was seen in MGYLS from preprocedure levels (1.78 ± 1.78) to the postprocedure one-week visit (3.75 ± 2.82, *p* < 0.0001). The improvement at one month remained significant (4.56 ± 2.85, *p* < 0.0001). Improvement was maintained at the three-month postprocedure visit (4.75 ± 3.08, *p* < 0.0001). There was no statistically significant difference between the one-week and the one-month visits (*p* = 0.687). Likewise, no difference was observed between the one-month and the three-month visits (*p* = 0.635) (Figure 8).

#### 3.2.2. Meibomian Gland Dropout

There were no statistical differences in meibomian gland dropout between baseline and three-month posttreatment of both the upper eyelid (*p* = 0.655) and the lower eyelid (*p* = 0.414). This suggests that treatment may not have an effect on the atrophic meibomian gland.

#### 3.2.3. Comparison of the Test Eye and the Control Eye

**Figure 9:**
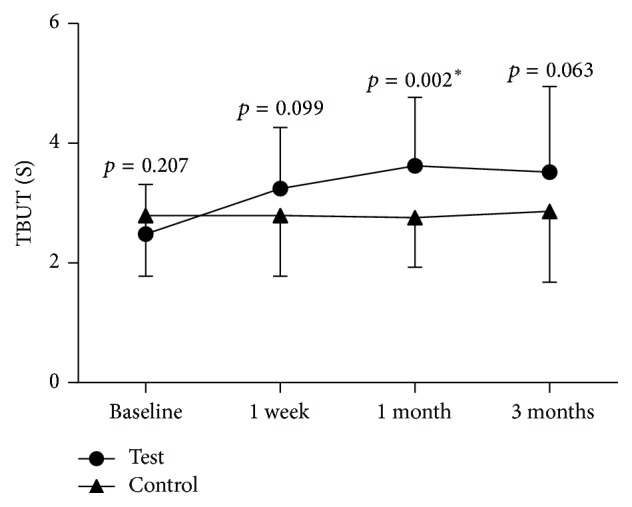
Time course of tear film breakup time (TBUT). Circle: test eye; triangle: control eye. There was a significant change between the test and control eye (*p* = 0.045). Differences of each point (one week, one month, and three months) posttreatment visit were labeled above the line (^*∗*^*p* < 0.05).

**Figure 10:**
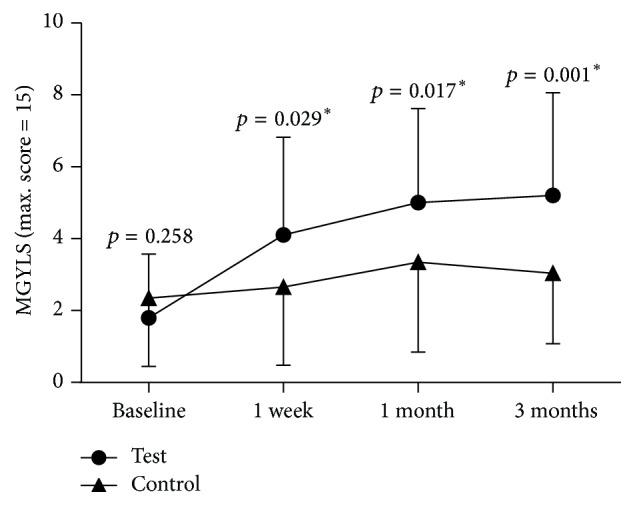
Time course of meibomian glands yielding liquid secretion (MGYLS). Circle: test eye; triangle: control eye. There was a significant change between the test and control eye (*p* = 0.019). Differences of each point (one week, one month, and three months) posttreatment visit were labeled above the line (^*∗*^*p* < 0.05).

Binocular baseline parameters are listed in Table 1. At baseline, there was no statistically significant difference for all measurements between control and test eyes (*p* > 0.05). After the treatment period, there were statistically significant changes in TBUT (Figure 9) and MGYLS (Figure 10).

During the posttreatment period, eight (28%) patients stopped using artificial tears and 11 (38%) patients reduced the frequency in which they use artificial tears. At the end of the three-month follow-up, only three patients reported that their subjective dry eye symptoms had not been improved. The remaining patients all reported improvements in their clinical symptoms at differing degrees.

## 4. Discussion

The goal of this study was to assess the safety and effectiveness of a 12-minute LipiFlow monocular treatment on elderly Chinese patients with MGD. Previous studies reported that a single LipiFlow 12-minute treatment improved meibomian gland secretion, TBUT, and symptom scores at one month, nine months, 12 months, and even three years posttreatment compared with traditional or new-style warm compress therapies and common clinical methods of physical expression of meibomian gland obstruction [16].

Previous studies evaluated the efficacy of LipiFlow by treatment of both eyes. Treatment of both eyes considerably increases the cost for patients in developing countries. This is the first study to report the results of monocular treatment with LipiFlow with the contralateral eye as a control.

The primary clinical parameters used to assess the effectiveness of LipiFlow indicated significant improvement of meibomian gland function and tear film stability in the test eye. These results demonstrate that treatment restored the function of previously blocked dysfunctional meibomian glands and an increase in gland function improved tear film stability, which may directly influence other objective and subjective measures of ocular surface health. Briefly, the prolonged TBUT, reduced corneal staining, and improved subjective symptoms provide strong evidence of the efficiency of LipiFlow.

However, significant changes of LLT were not observed. Previous studies report the repeatability and reliability of LLT measured by Lipiview [17]. Finis et al. [18] found that LLT increased significantly six months after treatment with LipiFlow. During the follow-up period, we observed that the consistency of meibum secreted from the meibomian gland greatly influenced LLT. Unhealthy meibum, which has a high LLT value, may have altered the results of our study. We plan to focus on the relationship between the consistency of meibum and LLT in future studies. Interestingly, we observed that Schirmer I test results, which represents tear fluid secretion, began to decrease at one week and continued to decrease at the three-month posttreatment examination. We speculate that the reopening of the meibomian glands stabilizes the tear film, making the tear fluid reflexively decrease to maintain homeostasis at the ocular surface. The existence of a homeostatic state of the tear film was proposed by Dartt and Willcox [19] where components of the tear film may compensate for deficiencies in others. Arita et al. [20] reported that an increase in tear fluid production likely compensates for loss of meibomian glands in MGD patients. Our results support the hypothesis of the homeostatic nature of the tear film.

Blink is an action associated with consciousness [21]; this behavior is difficult to change in a short time. Furthermore, during the examination of the partial blink by Lipiview, there is a strong flicker which could disturb the patients blink in nature. These may explain why partial blink was unchanged before and after treatment. We will make further explorations about the association between blink and dry eye.

Our study reports improvement in the subjective symptoms of control eye, which did not receive treatment, after three months of the monocular treatment. We speculate that this could be attributed to the placebo effect. Placebos are known to have an effect on the immune system [22]. It has been suggested that the effectiveness of a treatment or placebo is shaped by the subjects' expectations [23]. For example, patients with dry eye have increased incidence of depression and anxiety [24, 25]. Treatment, even placebo, may improve their clinical outcome and may explain why treatment in one eye has an effect on both eyes.

In this study, we excluded patients with severe meibomian gland atrophy (more than fifty percent) as they may respond poorly to treatment. Consistent with previously published studies, meibomian gland morphology had no obvious change posttreatment. This might also explain why patients with early stage of MGD are more likely to respond positively to this treatment. It is also possible that three months may not be long enough to detect changes in meibography as the regrowth of new glands and ducts may take longer.

In conclusion, our study suggests that the eyes treated by LipiFlow improved dramatically in objective parameters compared with contralateral eye in patients with MGD. Furthermore, monocular treatment can be beneficial to improve the subjective symptoms of those afflicted with MGD. Monocular treatment with LipiFlow may be a cost-effective alternative for treatment of MGD in developing countries.

## Figures and Tables

**Table 1:** Baseline parameters.

	Lipiview (ave.)	Lipiview (max.)	Lipiview (min.)	Partial blink^*∗*^	BUT^*∗*^	Schirmer^*∗*^	Meibomian gland orifices^*∗*^	Corneal stain^*∗*^
Test	62.07 ± 23.98	75.76 ± 21.61	54.24 ± 23.79	0.65 ± 0.35	2.48 ± 0.83	8.17 ± 6.96	1.79 ± 1.78	1.21 ± 0.62
Control	68.90 ± 23.32	77.93 ± 20.03	59.59 ± 22.61	0.70 ± 0.33	2.79 ± 1.01	8.76 ± 6.42	2.34 ± 1.90	1.55 ± 0.74
Significance	0.052	0.516	0.194	0.778	0.119	0.560	0.130	0.309

^*∗*^Wilcoxon signed-rank test.

There were no significant differences for all of these measured parameters between the test and control eyes before treatment (*p* > 0.05).
